# Probing Genomic Diversity of *Cronobacter sakazakii* in the United States by Single Nucleotide Polymorphisms

**DOI:** 10.3390/foods15081306

**Published:** 2026-04-09

**Authors:** Wei Zhang, Catherine W. Y. Wong, Richard Zhang, Renmao Tian, Behzad Imanian, Yan Li, Hongmei Jiang

**Affiliations:** 1 Institute for Food Safety and Health, Illinois Institute of Technology, Bedford Park, IL 60501, USArtian@illinoistech.edu (R.T.); bimanian@illinoistech.edu (B.I.); 2 Hinsdale Central High School, Hinsdale, IL 60521, USA; rzhang7036@stu.hinsdale86.org; 3 Center for Research Informatics, Bioinformatics Core, University of Chicago, Chicago, IL 60637, USA; liyan@uchicagomedicine.org; 4 Department of Statistics and Data Science, Northwestern University, Evanston, IL 60208, USA; hongmei@northwestern.edu

**Keywords:** *Cronobacter sakazakii*, whole-genome sequencing (WGS), single nucleotide polymorphism (SNP), pan genome, phylogenetic reconstruction, genomic diversity, SNP thresholds, outbreak surveillance, population genomics

## Abstract

*Cronobacter sakazakii* is an opportunistic pathogen commonly associated with powdered infant formula and causes severe neonatal infections. While whole-genome sequencing (WGS)-based single nucleotide polymorphism (SNP) analysis has revolutionized surveillance and outbreak investigations, comprehensive population-level analyses remain limited, and establishing proper thresholds for detecting epidemiologically related *C. sakazakii* isolates requires assessment using large-scale genomic datasets. We analyzed 1870 *C. sakazakii* genomes from the United States (1970–2025) to examine pan- and core-genomic structure, analyze SNP distance matrices encompassing 1,747,515 unique pairwise comparisons, and reconstruct population phylogeny. Our analyses revealed exceptional genomic diversity with a large pan-genome of 24,035 gene families and an average of 29,442 ± 13,097 SNPs between genome pairs. Phylogenetic reconstruction identified 22 major clusters encompassing 89.3% of genomes, including environmental complexes demonstrating persistent contamination spanning multiple years. Using 209 monophyletic genome pairs with concordant metadata, we propose a tiered SNP threshold framework (≤234 to 506 SNPs) for detecting potentially epidemiologically-related genomes with improved sensitivity. As genomes from Michigan comprised 39.3% of the dataset, these thresholds should be interpreted with caution when applied to other US regions. This study provides population genomics infrastructure to enhance *C. sakazakii* surveillance and traceback studies for improving powdered infant formula safety.

## 1. Introduction

*Cronobacter sakazakii* is an opportunistic Gram-negative bacterial pathogen in the family *Enterobacteriaceae*, and can cause severe neonatal infections, including necrotizing enterocolitis, bacteremia, and meningitis, with case-fatality rates of about 40% [1,2,3,4,5,6]. This organism is frequently isolated from powdered infant formula (PIF) manufacturing environments and has been implicated in multiple high-profile product recalls, most notably the 2022 PIF recall that led to a nationwide shortage for months [7,8,9]. *C. sakazakii* possesses a ~4.4–4.6 Mb genome (56–57% G + C) [10,11] encoding virulence factors including outer membrane proteins, iron acquisition systems, and a type VI secretion system [10,12]. Sequence type 4 (ST4) predominates among neonatal meningitis cases [13], while ST1 is dominant in PIF facilities carrying extensive resistance gene repertoires [14,15].

Population genomics, the large-scale analysis of genomic variation across a species, is essential for understanding bacterial evolution, transmission dynamics, and calibrating epidemiological thresholds. Large-scale SNP-based studies of *Salmonella enterica* have elucidated some lineage-specific epidemiological patterns [16,17], population genomic frameworks for *Klebsiella pneumoniae* have mapped the global distribution of high-risk clones [18,19], and analyses of *Escherichia coli* and *Listeria monocytogenes* have revealed deep population structure and persistent contamination spanning years in food environments [20,21,22]. These studies demonstrate that understanding within-species diversity is essential for distinguishing outbreak clusters from background variations.

Despite some advances across *Enterobacteriaceae*, SNP-based genomic studies of *C. sakazakii* remain limited to small collections from individual outbreaks or specific facilities [23,24,25], without the population-scale sampling needed to characterize intraspecies diversity to establish robust SNP thresholds. This gap is critical given this pathogen’s ubiquitous nature, environmental persistence, extensive horizontal gene transfer, and ecological versatility that may lead to population dynamics distinct from other pathogens [12,13,26,27]. Molecular typing has advanced from MLST (>700 sequence types) [13] to cgMLST schemes [28] and some pan-genome analyses [29,30], yet comprehensive population-scale analysis is still lacking.

Whole-genome sequencing has revolutionized foodborne disease investigations, with US and international agencies now promoting WGS integration into routine disease surveillance and environmental monitoring [31,32,33]. However, the accuracy of SNP-based inference depends critically on the analytical method chosen, as different approaches can yield substantially different results from the same genomic data. Reference-based pipelines, including the CFSAN SNP Pipeline/CSP [34,35], Snippy [36], Lyve-SET [37], and Parsnp [38] are used to map query genomes against a single reference genome to identify high-confidence variant calls, but are inherently limited to genomic regions present in that reference [39,40,41,42,43]. In contrast, reference-free methods such as kSNP [44] and SKA2 [45] use k-mer decomposition strategies to eliminate reference genome bias and can detect variants across the entire pan-genome. Whole-genome alignment tools like MUMmer [46] offer additional approaches with less stringent computational frameworks. Each method involves different sensitivity-specificity trade-offs that can significantly influence SNP detection rates, phylogenetic reconstruction, and ultimately, epidemiological conclusions about strain relationships [41,47].

Benchmarking studies have consistently shown that different SNP discovery methods produce substantially different absolute counts from identical datasets [41,47]. These methods often vary by several-fold, but generally preserve the relative relationships between isolates [47,48]. This methodological variation has critical implications for surveillance practice: SNP thresholds calibrated with one method may not be reliably applied to results from another method [37,49,50]. Operational thresholds (e.g., <20–25 core-genome SNPs) have been applied in regulatory investigations of several foodborne bacterial pathogens, though these values were derived from limited outbreak datasets and have not been validated across diverse global populations [51,52,53]. While tiered threshold frameworks offering graded confidence levels have gained support [50], and recent modeling studies have proposed dynamic, species-specific approaches incorporating mutation rates and temporal parameters [54,55,56], no consensus exists on optimal methods or thresholds for detecting epidemiologically related strains [50,57]. This lack of standardization is compounded by the diversity of computational pipelines and analytical frameworks employed across laboratories worldwide [42,47,58].

To address these knowledge gaps, this study presents a comprehensive population genomic analysis of 1870 *C. sakazakii* genomes from the United States, which is the largest dataset analyzed for this species to date. We pursued four specific objectives: (1) characterize the pan-genome structure at the species level; (2) perform population-scale total SNP discovery to assess genomic diversity; (3) reconstruct phylogenetic population structure; and (4) develop population-based SNP thresholds for detecting epidemiologically related strains. Here we define epidemiologically related isolates as genome pairs that cluster phylogenetically and share concordant epidemiological metadata across three categories (isolation source, geographic location, and collection timeframe), indicating potential transmission links or common sources of contamination. By integrating phylogenetic evidence with epidemiological metadata, we establish context-appropriate thresholds designed to enhance *C. sakazakii* outbreak surveillance, source attribution studies, and root cause investigations.

## 2. Materials and Methods

### 2.1. Genome Dataset and Quality Control

A total of 1870 *C. sakazakii* genome assemblies from the United States were retrieved from NCBI GenBank (https://www.ncbi.nlm.nih.gov/genbank/, accessed 1 December 2025) in FASTA format with GCA_ accession numbers. Metadata including isolation source, geographic location, and collection date were extracted from BioSample records. Isolation sources were categorized as Environmental, Infant Formula, Infant Cereal, Clinical, Food/Other, or Unknown. Geographic locations were classified using state names, such as Michigan, Maryland, California, New York, Tennessee, Other USA, or Unknown. Collection dates spanned 1970–2025. Genome quality was assessed through a multi-step filtering pipeline applied to the full initial retrieval of 2974 *C. sakazakii* genome assemblies. As a first-pass filter, assembly-level statistics were evaluated for each genome, including total assembly size, GC content, contig count, and N50 value; assemblies falling outside the expected range for *C. sakazakii* (genome size 3.35–5.13 Mb; GC content 56.0–57.5%) were flagged for exclusion. Genome completeness and contamination were subsequently assessed using PPanGGOLiN v2.2.1 single-copy marker gene analysis [59], which evaluates gene content completeness, identifies redundant or chimeric sequences, and performs gene prediction validation across all input assemblies. This QC workflow was applied uniformly to all 2974 genomes. Assemblies failing completeness thresholds, exhibiting elevated contamination estimates, or showing evidence of fragmentation or assembly artifacts were excluded. Following this filtering process, 1104 low-quality genomes were removed, yielding a final curated dataset of 1870 high-quality genome assemblies that were used for all subsequent pan-genome, phylogenetic, and SNP analyses. For comparative reference, CheckM assessment data were available for 152 genomes as reported by individual submitters to NCBI; these independently confirmed high completeness (97.8 ± 1.2%) and low contamination (1.9 ± 1.1%), consistent with the quality criteria applied across the full dataset.

### 2.2. Pan-Genome Analysis

Pan-genome analysis was conducted using PPanGGOLiN v2.2.1 [59]. Protein-coding sequences were predicted using Prodigal v2.6.3 [60] with bacterial-optimized parameters. Predicted genes were clustered into homologous families using MMseqs2 v14.7e284 [61] with 80% identity and coverage thresholds. Gene families were partitioned using the Neighbors-Expectation Maximization algorithm into persistent or soft core (95–100% presence), shell (3–95% presence), and cloud (<3% presence) categories. Regions of genomic plasticity (RGPs) were identified as contiguous regions enriched in shell and cloud genes. Pan-genome openness was assessed using rarefaction analysis with 30 random subsampling iterations, and the growth parameter (γ) was calculated using Heaps’ law fitting.

### 2.3. SNP Discovery and Distance Matrix Construction

Total genome single nucleotide polymorphisms were identified using kSNP4.1 [44] with k-mer length k = 19, determined by Kchooser optimization. The reference-free k-mer approach eliminated potential reference selection bias. Pairwise SNP distances were calculated for all genome combinations, generating an 1870 × 1870 symmetric distance matrix. Matrix integrity was validated through symmetry verification and diagonal checks. Pairwise distances were categorized as Very Close (≤100 SNPs), Close (101–500 SNPs), Moderate (501–1000 SNPs), Distant (1001–5000 SNPs), or Very Distant (>5000 SNPs).

### 2.4. Phylogenetic Reconstruction

Maximum likelihood phylogenetic trees were constructed using RAxML v8.2.12 [62] with the GTRGAMMA substitution model [63]. Bootstrap confidence was assessed using 1000 replicates. Trees were midpoint-rooted for branch length balance. Major phylogenetic clusters were defined as monophyletic groups containing ≥50 genomes with bootstrap support ≥0.85.

Principal coordinate analysis (PCoA) was performed on the SNP distance matrix. Optimal population clustering was determined using K-means with silhouette score optimization. PERMANOVA testing evaluated associations between genomic structure and metadata variables. Temporal persistence was assessed by analyzing collection date ranges within clusters. Mantel tests evaluated correlations between genetic distance and temporal/spatial variables.

### 2.5. Evidence-Based Threshold Development

Epidemiologically related genome pairs were defined using three mandatory criteria: (1) monophyletic relationship (sister taxa in phylogenetic tree), (2) strong bootstrap support (>90%), and (3) metadata concordance (shared isolation source, geographic location, or collection year). Related pairs were stratified into confidence tiers: Tier 1 (all three metadata criteria concordant), Tier 2 (≥2 criteria concordant), Tier 3 (≥1 criterion concordant). Optimal SNP thresholds were determined using 95th percentile analysis for each tier to balance 95% sensitivity with specificity. Framework performance was compared against the FDA-proposed threshold (<21 SNPs) using sensitivity calculations.

### 2.6. Statistical Analysis and Computational Resources

Statistical analyses were performed using Python v3.10 [64] with NumPy v1.24 [65], pandas v1.5 [66], SciPy v1.11 [67], and scikit-learn v1.3 [68]; specialized correlation and agreement testing used pingouin v0.5.3 [69]. Descriptive statistics such as mean, median, standard deviation, range, coefficient of variation, and interquartile range, were computed for pairwise SNP distance distributions across all 1,747,515 genome comparisons. Because data distributions were non-normal, the Kruskal-Wallis H-test was used to compare SNP distances across sequencing/alignment methods, followed by pairwise Mann-Whitney U tests with Bonferroni correction (adjusted α = 0.005). Both Pearson and Spearman correlation coefficients were calculated to assess linear and monotonic relationships between methods, and agreement across methods was quantified using the Intraclass Correlation Coefficient (ICC, two-way random effects model). Population structure was characterized by Principal Coordinate Analysis (PCoA) on the full SNP distance matrix, and optimal genomic cluster number was determined by K-means clustering with silhouette score optimization. Associations between genomic structure and metadata variables (source, geography, collection year) were evaluated using PERMANOVA, while Mantel tests assessed correlations between genetic distance and collection date. Phylogenetic support was assessed using bootstrap values, with clusters requiring ≥85% bootstrap support for structural inference and >90% for high-confidence pair classification. SNP threshold performance for outbreak detection was evaluated by 95th-percentile analysis within confidence tiers, benchmarked against the FDA-proposed ≤21 SNP threshold using sensitivity calculations. All bioinformatics workflows were executed on a high-performance computing system (20-core Intel Xeon, 512 GB RAM), with complete source code, intermediate matrices, and metadata archived under version control to ensure full reproducibility.

#### Zero-Inflated Mixture Model for SNP Distance Distribution

To characterize the distribution of pairwise SNP distances among closely related genome pairs (<5000 SNPs), a zero-inflated three-component log-normal mixture model was fitted. This approach was necessary because the SNP distance data exhibited two features incompatible with standard parametric distributions: (1) an excess of exact zero values (~900 pairs, corresponding to identical genomes), and (2) a right-skewed, multimodal positive distribution reflecting discrete phylogenetic subpopulations within the closely related fraction of the dataset. A standard log-normal or Gaussian mixture without zero-inflation would misrepresent the true density at low SNP distances and thereby distort percentile-based threshold estimates. The model specifies that a pairwise SNP distance X follows:

X ~ π_0_ · δ_0_ + (1 − π_0_) · Σ_k=1_^3^ w_k_ · LogNormal(μ_k_, σ_k_^2^)

where δ_0_ is a degenerate point mass at zero (assigning probability 1 to X = 0), π_0_ is the estimated probability of an exact zero, w_k_ are the mixture weights for the three log-normal components (with Σw_k_ = 1), and μ_k_ and σ_k_^2^ are the mean and variance of the log-transformed distances within each component.

Parameter estimation was carried out in two steps. First, the zero-inflation probability π_0_ was estimated empirically as the proportion of exact zero distances in the dataset. Second, conditional on positive distances (X > 0), the three-component log-normal mixture parameters {w_k_, μ_k_, σ_k_^2^}_k=1_^3^ were estimated using the Expectation–Maximization (EM) algorithm. All analyses were performed in R v4.5.2 [70] using the mclust package [71], which implements maximum likelihood estimation for Gaussian mixture models via the EM algorithm.

Although the Bayesian Information Criterion (BIC) is the conventional criterion for selecting the number of mixture components, it consistently favored models with a larger number of components in this dataset. This behavior is expected given the very large number of pairwise distances (n > 300,000 pairs with SNP distance < 5000) and the well-known tendency of mixture models to exploit additional components to approximate fine-grained distributional features rather than biologically meaningful subgroups. Accordingly, BIC was not used as the sole criterion for model selection. Instead, a parsimonious three-component model was adopted to provide an interpretable and biologically grounded stratification of SNP distances among closely related genome pairs. The three components capture increasing levels of genetic divergence within this closely related fraction: pairs that are nearly identical (very small SNP distances), extremely close but not identical, and moderately close. This stratification directly aligns with the operational goal of characterizing fine-scale genetic similarity in the context of outbreak and contamination source attribution, while avoiding overfitting to noise in the distance distribution.

To confirm that the key quantitative summaries are robust to the choice of the number of components, we compared percentile estimates under K = 3 and K = 5. Under K = 3, approximately 1% of pairwise SNP distances among closely related pairs fall below 2 SNPs, 5% below 22 SNPs, and 10% below 207 SNPs, with a median of 757 SNPs and a 75th percentile of 1138 SNPs. Under K = 5, the corresponding estimates are: 1% below 2 SNPs, 5% below 23 SNPs, 10% below 215 SNPs, a median of 754 SNPs, and a 75th percentile of 1150 SNPs. The negligible differences between these two models confirm that the reported percentile thresholds are not sensitive to the choice of K, supporting the validity of the three-component model as the basis for the tiered SNP threshold framework.

## 3. Results

### 3.1. Dataset Composition and Characteristics

**Figure 1:**
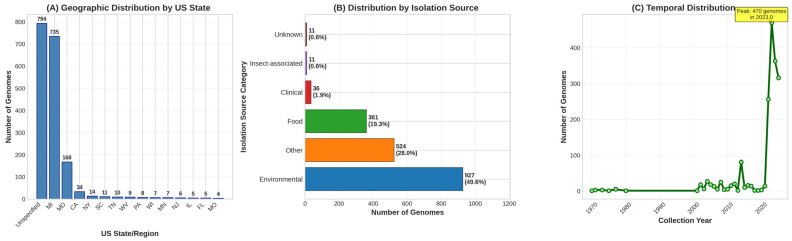
Geographic and source distribution of 1870 *Cronobacter sakazakii* Genomes. (**A**) Geographic distribution by US state. Michigan contributed the largest number of genomes (735, 39.3%), followed by unspecified U.S. locations (794, 42.5%) and Maryland (168, 9.0%). The remaining genomes were distributed across 39 additional states. (**B**) Distribution by isolation source. Environmental samples dominated (927, 49.6%), followed by other/unspecified sources (524, 28.0%), food-related sources (361, 19.3%), clinical specimens (36, 1.9%), and insect-associated isolates (11, 0.6%). (**C**) Temporal distribution. Genome collection from 1970–2025 showing sparse early sampling followed by exponential growth after 2020 with widespread adoption of whole genome sequencing. Peak collection occurred in 2023 (470 genomes).

A comprehensive dataset of 1870 *C. sakazakii* genomes was assembled from NCBI databases, representing the largest *C. sakazakii* collections analyzed to date (Figure 1). All genomes originated from the United States with broad geographic representation across 42 states, though distribution was heterogeneous with Michigan contributing the largest proportion (735 genomes, 39.3%) followed by unspecified U.S. locations (794 genomes, 42.5%). The temporal span covered 55 years (1970–2025), with dramatic increases in genome availability after 2020 coinciding with widespread adoption of whole genome sequencing for surveillance.

Assembly quality was assessed across all 1870 genomes using the PPanGGOLiN-based QC pipeline described in Section 2.1. These 1870 assemblies represent the high-quality subset retained from an initial pool of 2974 genomes, following the exclusion of 1104 assemblies that failed completeness, contamination, or assembly integrity thresholds. Mean assembly size was 4.57 ± 0.13 Mb (range: 3.35–5.13 Mb) and GC content was 56.9 ± 0.2% (range: 56.0–57.5%), consistent with published *C. sakazakii* genome characteristics. The majority of assemblies were contig-level (1833, 98.0%), with scaffold-level (33, 1.8%) and complete genomes (4, 0.2%) also represented. For 152 genomes for which CheckM assessment data were available from NCBI submitters, mean completeness was 97.8 ± 1.2% and mean contamination was 1.9 ± 1.1%, independently corroborating the quality of the curated dataset. The taxonomic homogeneity, extensive temporal and geographic coverage, and diverse epidemiological contexts provide an ideal foundation for investigating *C. sakazakii* evolutionary patterns and population structure.

### 3.2. Pan-Genome Architecture and Core Genome Characteristics

**Figure 2:**
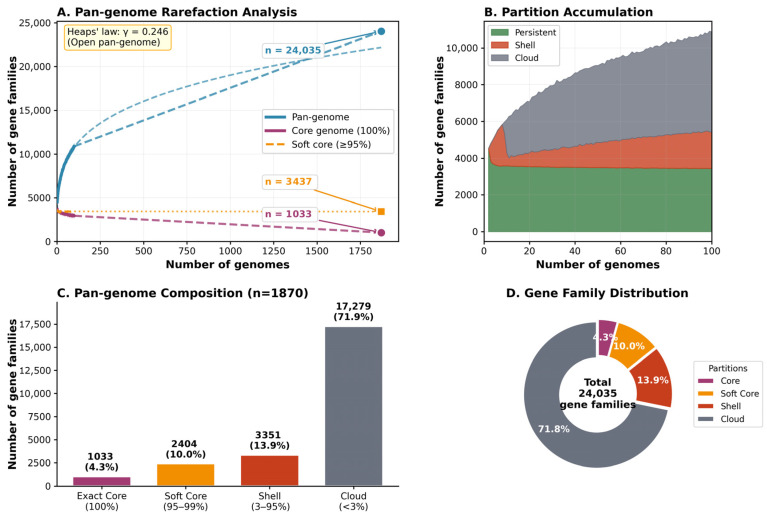
Pan-genome analysis of *Cronobacter sakazakii* (n = 1870 genomes) (**A**) Pan-genome rarefaction analysis shows pan-genome expansion (blue line, n = 24,035 gene families) and core genome contraction with increasing sample size. Heaps’ law fitting (γ = 0.246) confirms an open pan-genome structure with continued gene discovery. Core genome (100% presence) contains 1033 families, while soft core (≥95% presence) contains 3437 families. (**B**) Partition accumulation. Stacked area plot showing the accumulation of PPanGGOLiN partitions (persistent, shell, cloud) as genomes are added, demonstrating the dominance of cloud genes in driving pan-genome expansion. (**C**) Pan-genome composition. Bar chart showing the distribution of 24,035 gene families across partitions: exact core (1033, 4.3%), soft core (2404, 10.0%), shell (3351, 13.9%), and cloud (17,279, 71.9%). (**D**) Gene family distribution. Pie chart visualizing the proportional composition of pan-genome partitions, highlighting the predominance of cloud genes (71.8%) representing rare, strain-specific content.

Pan-genome analysis of 1870 *C. sakazakii* genomes yielded 24,035 gene families from 7,855,496 predicted protein-coding sequences (Figure 2). PPanGGOLiN’s statistical partitioning classified these into three compartments: persistent (3405 families, 14.2%), shell (3351 families, 13.9%), and cloud (17,279 families, 71.9%) (Table 1). The strict core genome (present in 100% of genomes) comprised only 1033 gene families (4.3%) and represented one of the smallest core genomes documented among *Enterobacteriaceae*. The percentage of soft core genome (14.3%) of *C. sakazakii* was comparable to those of *Salmonella enterica* (12.6%) and *Escherichia coli* (12%) (Table 2). Using the soft core definition (≥95% presence), the core expanded to 3437 families (14.3%), suggesting approximately 2400 additional families are nearly universal but absent in only a small subset of strains due to gene loss, pseudogenization, or assembly artifacts. The percentage of soft core genome estimation in this study (14.3%) based on 1870 *C. sakazakii* genomes was smaller than those in two previous studies (19.5% and 25.2%) based on 237 [29] and 748 [30] genomes, respectively, largely due to the different pan-genomes identified (Table 2).

The dominant accessory genome (85.8%) reflects extensive genomic plasticity driven by horizontal gene transfer. Analysis identified 81,495 Regions of genomic plasticity (RGPs) across all genomes (mean: 43.6 ± 8.7 per genome), with 153 integration hotspots where ≥50% of RGPs from different genomes overlapped, indicating conserved preferential sites for horizontal gene acquisition. Additionally, 922 functional modules containing 5525 gene families were identified, representing co-localized gene clusters potentially transferred as coordinated units.

Rarefaction analysis confirmed an open pan-genome following Heaps’ law (γ = 0.246), indicating that sequencing additional genomes would continue to reveal novel gene families. Compared with two previous studies [29,30], our expanded dataset revealed 6877 to 10,272 additional gene families while the soft core genome remained largely unchanged. Individual genomes contained an average of 4096 gene families (SD: 167), distributed as ~3380 persistent, ~580 shell, and ~140 cloud genes per genome.

### 3.3. SNP-Based Population Genomic Analysis

**Figure 3:**
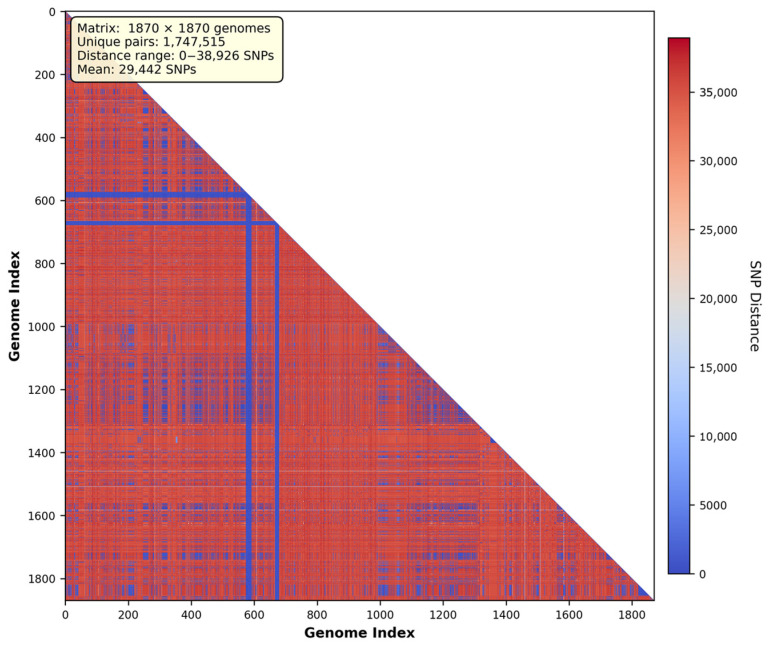
SNP distance matrix—triangular view. Triangular heatmap displaying pairwise SNP distances among 1870 *Cronobacter sakazakii* genomes. The matrix contains 1,747,515 unique pairwise comparisons with SNP distances ranging from 0 to 38,926 substitutions (mean: 29,442 SNPs). Color scale represents SNP distance from blue (low divergence, 0 SNPs) to red (high divergence, >35,000 SNPs). Distinct blue blocks indicate closely related genome clusters, while the predominant red coloration reflects extensive genetic diversity within the species. Vertical and horizontal blue lines at approximately genome index 600 highlight a major phylogenetic division, corresponding to the Michigan environmental complex identified in subsequent phylogenetic analysis.

Pan-genome SNP analysis using kSNP4.1 identified a total of 891,621 SNP sites across the 1870-genome dataset (Figure 3). Of these, 159,020 SNP sites (17.83%) were located in the soft core genome (present in ≥95% of genomes). The resulting SNP distance matrix contained 1,747,515 unique pairwise comparisons, with SNP distances ranging from 0 to 38,926 substitutions (mean: 29,442 ± 13,097; median: 35,278) (Figure 3). Distance distribution was highly skewed (Table 3). Combined, epidemiologically relevant comparisons (≤500 SNPs) represented only 4.35% of all relationships, indicating limited clonal expansion within the sampled population.

**Figure 4:**
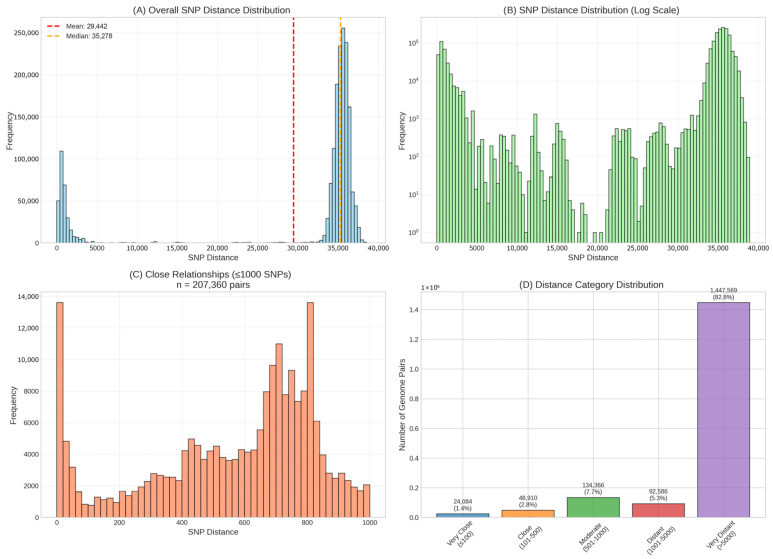
SNP distance distribution analysis. (**A**) Histogram shows the distribution of 1,747,515 unique pairwise SNP distances with mean 29,442 SNPs (red dashed line) and median 35,278 SNPs (orange dashed line). The bimodal distribution reflects distinct population structure with peaks representing intra- and inter-lineage comparisons. (**B**) Log-transformed frequency distribution reveals the full range of genetic diversity, from identical genomes (0 SNPs) to highly divergent strains (>35,000 SNPs). The log scale highlights the relative frequency of close relationships amid the predominantly distant comparisons. (**C**) Detailed view of 207,360 genome pairs with SNP distances ≤1000. The multimodal pattern indicates discrete phylogenetic clusters with varying internal diversity. (D) Categorical breakdown of all pairwise comparisons: identical pairs, very close relationships 1–100 SNPs, close relationships 101–500 SNPs, moderate distances 501–1000 SNPs, and distant or very distant relationships >1000 SNPs.

The histogram of pairwise SNP distances in Figure 4 shows a clear bimodal structure, indicating the presence of two distinct clusters. The cluster concentrated near zero corresponds to pairs of closely related samples with small genetic distances, while the cluster on the right corresponds to pairs of genetically distant samples with large SNP distances.

We further focused on the analysis of genome pairs with <5000 SNPs. Let X denote the pairwise distance. Noticing that there are about 900 values of X equal to 0, we model X using a zero-inflated mixture distribution to accommodate excess zeros and heterogeneous positive values. Specifically, X is assumed to follow a mixture of a point mass at zero with probability π_0_ and a three-component log-normal mixture for positive values with probability $1-\pi_{0}$:

$$X∼\pi_{0}\delta_{0}+\left(1-\pi_{0}\right)\sum_{k=1}^{3}w_{k}\;\mathrm{LogNormal}\left(\mu_{k},\sigma_{k}^{2}\right).$$

Here $\delta_{0}\;$ denotes a degenerate distribution at zero, i.e., it assigns probability 1 to the value X = 0 and 0 elsewhere. The mixture weights satisfy $\sum_{k=1}^{3}w_{k}=1$, and $\mu_{k}\;$ and $\sigma_{k}^{2}\;$ represent the mean and variance of the log-transformed positive observations within each component. This formulation allows flexible modeling of skewed positive values while explicitly accounting for zero inflation. Using this mixture model, among the closely related samples, 1% of pairwise SNP distances fall below 2, 5% below 22, 10% below 207, with a median of 757 and a 75^th^ percentile of 1138. Sensitivity analysis comparing K = 3 and K = 5 component models confirmed that these percentile estimates are robust to the choice of the number of components, with differences of ≤8 SNPs across all reported quantiles (see Section “Zero-Inflated Mixture Model for SNP Distance Distribution” for details).

Principal coordinate analysis explained 65.74% of total variance in the first five dimensions (PC1: 29.33%, PC2: 16.10%, PC3: 7.18%, PC4: 7.05%, PC5: 6.08%), revealing distinct population structure with clear genetic differentiation among major lineages. K-means clustering optimization identified k = 6 as optimal with a silhouette score of 0.895, indicating well-defined population structure.

PERMANOVA testing revealed significant associations between genomic variation and epidemiological metadata: isolation source (F = 19.70, ρ = 0.001, Cramér’s V = 0.380), geographic state (F = 12.08, ρ = 0.001, Cramér’s V = 0.359), and host category (F = 3.03, ρ = 0.003, Cramér’s V = 0.427), all with large effect sizes. Temporal analysis via Mantel testing showed no correlation between genetic distance and collection time (r = 0.0139, ρ = 0.316). Cluster validation identified one cluster with a within/between cluster distance ratio of 3.44 (Mann-Whitney U test: ρ < 0.001), indicating significantly tighter genetic clustering within the cluster compared to external genomes.

### 3.4. Phylogenetic Reconstruction and Population Structure

**Figure 5:**
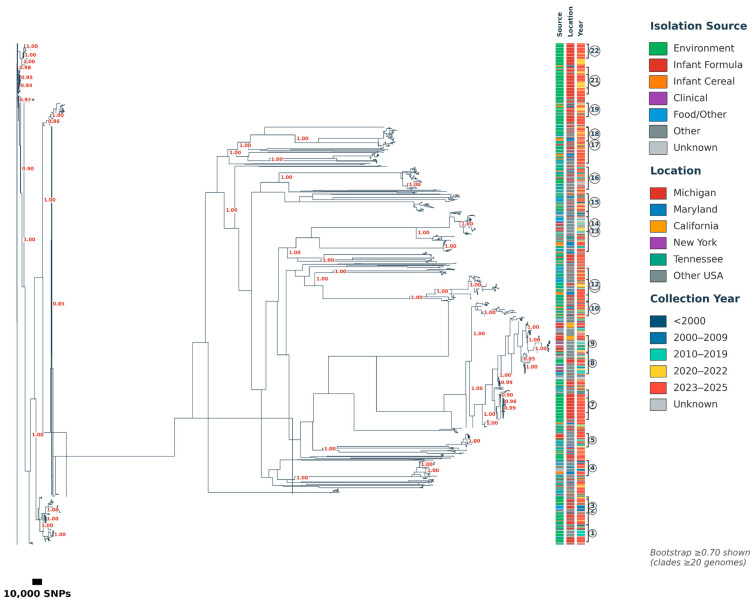
Maximum likelihood phylogenetic tree of 1870 *Cronobacter sakazakii* genomes constructed from 891,621 total SNPs, revealing 22 major clusters (≥50 genomes each, numbered 1–22). Bootstrap support values ≥0.85 are shown in red at key nodes. Colored strips represent isolation source (green: environmental, red: infant formula, orange: infant cereal, purple: clinical, blue: food/other, gray: other/unknown), geographic location (red: Michigan, blue: Maryland, orange: California, purple: New York, teal: Tennessee, gray: other USA), and collection year (dark blue: <2000, teal: 2000–2009, light blue: 2010–2019, yellow: 2020–2022, orange/red: 2023–2025, gray: unknown). Scale bar represents 10,000 SNPs.

Maximum likelihood phylogenetic analysis using 891,621 total SNPs revealed 22 major clusters containing ≥50 genomes each, encompassing 1669 isolates (89.3% of the dataset) (Figure 5). Bootstrap support exceeded 0.90 for 18 of 22 clusters (81.8%), with 14 clusters (63.6%) achieving perfect support (1.00), confirming robust phylogenetic relationships (Table 4).

**Figure 6:**
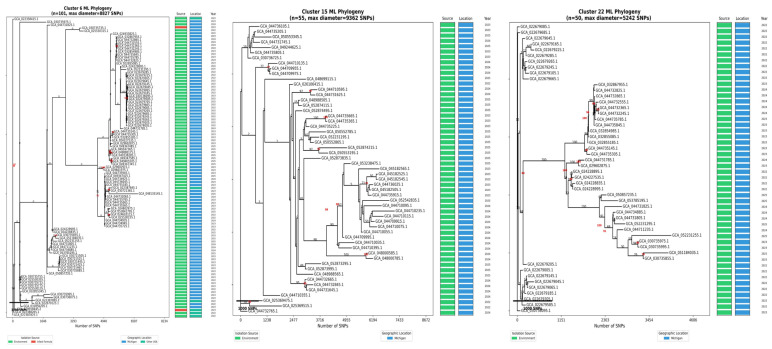
Maximum likelihood phylogenetic trees of three representative clusters from the Michigan environmental complex, demonstrating persistent environmental contamination patterns. Bootstrap support values ≥ 0.7 are shown in red at nodes. Colored bars indicate isolation source (green: environmental; red: infant formula), geographic location (blue: Michigan), and collection year. Scale bars represent SNP distances.

The most prominent finding was a Michigan environmental complex comprising five major clusters (>80% of genomes from Michigan in clusters 4, 6, 15, 17, 22) containing 475 genomes (25.4% of all isolates), demonstrating temporal persistence spanning 24 years (2001–2025) and strong geographic clustering (475/735 Michigan isolates, 64.6%). Three representative clusters are shown in Figure 6. Cluster 6 (n = 101, max diameter = 8827 SNPs) consisted of predominantly environmental isolates (98.0%) from Michigan (94.1%) spanning 2022–2025, showing recent emergence with tight genetic clustering and sustained contamination over multiple years. Cluster 15 (n = 55, max diameter = 9362 SNPs) consisted of exclusively environmental isolates (100%) from Michigan (100%) collected 2022–2025, representing a geographically and temporally focused contamination event with moderate internal genetic diversity. Cluster 22 (n = 50, max diameter = 5242 SNPs) consisted of all environmental isolates (100%) from Michigan (100%) spanning 2022–2025, showing the most genetically homogeneous cluster with the smallest maximum diameter and indicating recent common ancestry and ongoing transmission. These clusters collectively demonstrate the persistence and genetic stability of *C. sakazakii* populations in Michigan PIF production environments. Infant formula-associated clusters showed distinct patterns: Cluster 11 (62 genomes, 66.1% formula association, broad U.S. distribution) and Cluster 19 (51 genomes, 41.2% formula association). Clinical isolates (n = 42) distributed across multiple clusters rather than forming distinct clades, supporting diverse infection sources and multi-reservoir exposure pathways.

This phylogenetic framework establishes baseline diversity expectations for genomic surveillance, enabling rapid contextualization of new isolates within the population structure and identification of potential transmission routes and cross contamination scenarios.

### 3.5. Evidence-Based SNP Threshold Framework Development

From 1870 *C. sakazakii* genomes, we identified 372 monophyletic pairs with bootstrap support >90%. Of these, 209 pairs (56.2%) met at least one metadata concordance criterion (shared isolation source, geographic location, or collection year) (Table 5). Stratification by concordant criteria yielded three tiers: Tier 1 (met all three criteria, n = 110), Tier 2 (met two criteria, n = 59), and Tier 3 (met one criterion, n = 40).

SNP distance distributions differed significantly between tiers (Kruskal-Wallis ρ < 0.001), with Tier 1 showing the lowest median distance (3 SNPs, 95th percentile: 234), followed by Tier 2 (median 7 SNPs, 95th percentile: 506) and Tier 3 (median 10 SNPs, 95th percentile: 498). All tiers displayed substantial ranges extending to 1128 SNPs, reflecting the complex relationship between epidemiological concordance and genetic similarity.

**Figure 7:**
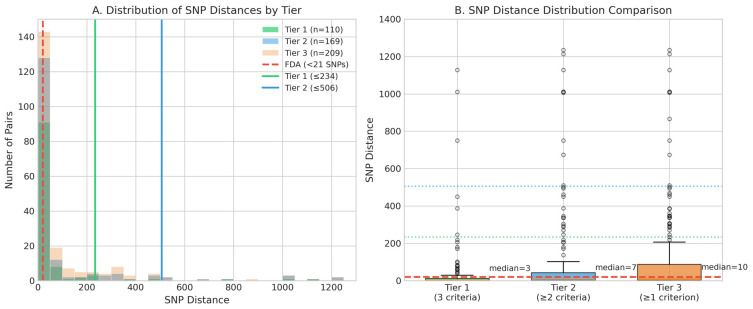
SNP distance distributions and tiered threshold framework. (**A**) Histogram showing SNP distance distributions for monophyletic genome pairs classified into three tiers based on metadata concordance. Tier 1 (green, n = 110) represents pairs with all three criteria matching (source + location + year), Tier 2 (blue, n = 169) has ≥2 criteria matching, and Tier 3 (orange, n = 209) has ≥1 criterion matching. Vertical lines indicate current FDA threshold (<21 SNPs, red dashed line) and proposed tiered thresholds: Tier 1 (≤234 SNPs, green solid line) and Tier 2 (≤506 SNPs, blue solid line). Most epidemiologically concordant pairs cluster at low SNP distances, but substantial ranges extend to >1000 SNPs across all tiers. (**B**) Box plots comparing SNP distance distributions across the three tiers. Tier 1 shows the lowest median (3 SNPs), followed by Tier 2 (median = 7 SNPs) and Tier 3 (median = 10 SNPs). Gray dotted lines indicate the 95th percentile thresholds used for the tiered framework (234, 506, and 498 SNPs respectively). The FDA threshold (<21 SNPs, red dashed line) captures only a fraction of epidemiologically related pairs, while the proposed tiered thresholds achieve 95% sensitivity across all confidence levels.

Performance comparison (Figure 7) revealed insufficient sensitivity of the FDA threshold (<21 SNPs): Tier 1 (76.4%), Tier 2 (66.3%), and Tier 3 (58.9%). The proposed tiered framework using 95th percentile thresholds (≤234 to 506 SNPs) achieved 95% sensitivity across all categories. Validation using the Michigan environmental cluster complex confirmed framework performance: all within-cluster comparisons from Clusters 15, 17, and 22 fell below Tier 1 thresholds, while the FDA threshold would have missed 67–89% of these relationships.

## 4. Discussion

### 4.1. Genomic Architecture and Evolutionary Implications

The pan-genome of *C. sakazakii* in this study comprised 24,035 gene families from 7,855,496 predicted genes, and was significantly larger than previously reported pan-genomes of this species [29,30]. The dominance of cloud genes (71.9%) indicates extensive strain-specific content reflecting ongoing horizontal gene transfer and niche adaptation, while the open pan-genome structure (γ = 0.246) confirms continued evolutionary diversification consistent with ecological plasticity across diverse environmental and clinical niches.

The identification of 81,495 RGPs with 153 integration hotspots provides mechanistic insight into genomic plasticity, aligning with the organism’s remarkable environmental persistence and pathogenic versatility. This genomic flexibility likely facilitates survival across conditions ranging from powdered infant formula manufacturing environments to neonatal bloodstream infections. Comparative analysis positions *C. sakazakii* as exhibiting similar genomic diversity to *E. coli* and *S. enterica*, potentially reflecting dual lifestyle requirements for environmental persistence and virulence with the small core genome representing essential functions while the extensive accessory genome enables niche-specific adaptation.

### 4.2. Population Structure and Epidemiological Significance

The extensive genetic diversity observed (mean 29,442 ± 13,097 SNPs) substantially exceeds values reported for many foodborne bacterial pathogens, indicating deep evolutionary divergence within the species. The skewed distance distribution, with 94.4% of comparisons showing >1000 SNPs, demonstrates limited recent clonal expansion and supports SNP-based outbreak investigation utility.

Principal coordinate analysis revealed structured population genetics with 65.74% variance explained in five dimensions, reflecting non-random genetic associations. Significant PERMANOVA associations with isolation source (F = 19.70, *p* = 0.001) and geographic location (F = 12.08, *p* = 0.001) confirm that genetic structure correlates with epidemiological metadata, validating the biological relevance of phylogenetic clustering.

The identification of a Michigan environmental complex comprising 475 genomes (25.4% of the dataset) with 24-year persistence represents compelling evidence for long-term environmental contamination in food production facilities. This finding has immediate regulatory implications, suggesting that environmental monitoring and facility decontamination protocols may require enhancement to address persistent *C. sakazakii* populations.

### 4.3. Population-Based Tiered Threshold Framework

Current foodborne pathogen outbreak surveillance relies predominantly on single-threshold genetic distance approaches, exemplified by the FDA’s < 21 SNP cutoff derived from a limited set of acute outbreak investigations [51]. Such thresholds may lack sufficient sensitivity to detect epidemiologically related isolates that have diverged beyond the threshold boundary despite sharing a common contamination source [50,55]. In the present study, the FDA’s < 21 SNP criterion captured only a fraction of epidemiologically related isolates, failing to identify 67–89% of putative relationships within the Michigan environmental complex. This limited sensitivity is an expected consequence of deriving thresholds from acute outbreak scenarios, which may not adequately represent the broader genomic diversity characterizing persistent environmental contamination, particularly in facilities where multiple genetically distinct strains co-circulate over extended timeframes, a pattern well-documented for *C. sakazakii* in powdered infant formula manufacturing environments.

A uniform single-threshold framework is further constrained by its inability to accommodate the varying epidemiological evidence and operational objectives inherent to different surveillance contexts [50,55]. Outbreak confirmation, investigational follow-up, and broad environmental monitoring represent distinct applications with fundamentally different requirements for sensitivity and specificity; applying identical genetic distance criteria across these contexts conflates their distinct objectives and risks both over- and under-attribution of strain relatedness [57].

Traditional criteria for classifying isolates as “epidemiologically related” have been shaped predominantly by acute outbreak investigations, in which relationships are established through confirmed illness clusters, case-control studies, and product traceback [51,77]. While this framework is appropriate for acute public health responses, its outbreak-centric focus can create systematic surveillance blind spots because it recognizes strain relationships only when linked to detected cases of human illness [78,79,80]. This framework can also overlook relatedness among isolates sharing a common contamination event that has not yet or may never manifest as a recognized outbreak [31,81,82].

To address this limitation, we propose expanding the definition of epidemiological relatedness to encompass broader patterns of epidemiological concordance. Our metadata-concordance framework operates on the premise that isolates sharing coherent characteristics across isolation source, geographic location, and temporal window may represent epidemiologically meaningful relationships even in the absence of confirmed outbreak linkage, including common contamination events, persistent environmental reservoirs, or transmission pathways not yet associated with detected illness.

This expanded definition serves three critical surveillance needs that outbreak-centric frameworks cannot address [77,78,79]. First, it enables identification of contamination sources before they manifest as clinical cases, supporting preventive intervention [77,83]. Many contamination events represent “silent” food safety risks that produce no detected illness due to low infectious doses, limited exposure of vulnerable populations, or surveillance gaps [79]. Second, it facilitates detection of persistent environmental contamination within production facilities, particularly relevant for *C. sakazakii* given its well-characterized desiccation tolerance and biofilm formation capacity [84,85]. Third, it accommodates incomplete epidemiological metadata by leveraging available information rather than requiring perfect documentation for a relationship to be recognized [31,49,86,87].

Our population genomics-based tiered framework addresses these limitations by providing context-appropriate guidance calibrated to different levels of epidemiological confidence. The framework stratifies relationships into three tiers: Tier 1 (≤234 SNPs) for detecting potential epidemiologically-related genomes requiring further epidemiological investigation and support when isolates share all three metadata criteria; Tier 2 (≤506 SNPs) and Tier 3 (≤498 SNPs) for supporting broader surveillance monitoring when isolates share limited criteria. Validation using phylogenetically confirmed clusters demonstrated substantial improvements over current approaches, with sensitivity gains of 18.6–36.1% across all confidence levels relative to FDA thresholds. Validation against the Michigan environmental complex was particularly informative: all within-cluster comparisons fell below Tier 1 thresholds, confirming the framework’s capacity to capture epidemiological relationships that single-threshold approaches systematically miss.

This tiered structure balances sensitivity and specificity across the full range of surveillance applications, from acute outbreak response to long-term environmental monitoring. By providing epidemiologically informed, statistically validated thresholds, the framework supports more effective contamination source identification, facility persistence monitoring, and regulatory decision-making support, pending prospective validation with confirmed outbreak data, with direct implications for protecting vulnerable infant populations.

### 4.4. Study Limitations and Implications for Food Safety Surveillance

This study, while representing the largest *C. sakazakii* genomic dataset to date (n = 1870), has several limitations affecting generalizability. The dataset contains exclusively U.S. isolates, which may not fully represent global genomic diversity, and geographic concentration in Michigan (39.3%) and Maryland (9.0%) introduces potential bias in population structure interpretations. The disproportionate representation of Michigan isolates is largely due to recently enhanced environmental monitoring conducted in that region, and threshold estimates may therefore be influenced by this sampling structure.

We used kSNP4 as a reference-free approach for large-scale phylogenomic analysis. The k-mer based methodology, though effective at eliminating reference bias, may classify positions containing gaps, ambiguous nucleotides (Ns), or assembly artifacts as legitimate SNPs when k-mers are detected in sufficient genomes [20,44]. In large, diverse datasets such as our 1870 *C. sakazakii* genomes spanning multiple highly divergent lineages and various assembly qualities, 77.4% of SNP positions exhibited some missing data across the dataset, reflecting the substantial genomic heterogeneity inherent to intra-species collections [88]. These missing data may inflate evolutionary distances between distantly related genomes and introduce noise into SNP distance calculations, particularly in inter-lineage comparisons where assembly differences are prevalent [41,47]. Consequently, phylogenetic tree topology and epidemiologically relevant SNP thresholds should be interpreted with these dataset characteristics in mind [37,50]. Core genome approaches that restrict SNP calling to universally present, high-quality genomic regions, or the application of stricter data completeness filters, represent complementary strategies for obtaining more conservative estimates of evolutionary relationships [88,89].

Limited metadata availability presents additional constraints. Many isolates in the NCBI database lack detailed source, location, or temporal information, a gap that is particularly pronounced for historical isolates and that reduces precision in epidemiological relationship assessments [57,86,90,91]. Most critically, the absence of validated outbreak linkages between environmental, food, and clinical isolates necessitates reliance on phylogenetic inference and metadata concordance as proxies for true epidemiological relatedness [55]. While the tiered framework provides statistically robust thresholds, this absence of ground-truth validation reduces confidence in threshold precision and represents an inherent limitation of any genomic surveillance framework developed in the absence of prospectively documented outbreak data [37,50,57,82].

This reflects a broader challenge in *C. sakazakii* surveillance: documented outbreaks are rare relative to apparent environmental prevalence, creating fundamental difficulties in establishing evidence-based thresholds through conventional epidemiological validation [26,55,74,92,93]. Future studies incorporating real-time outbreak investigations, ideally with matched environmental, food product, and clinical isolates, would enable empirical threshold refinement and strengthen the evidentiary basis for regulatory adoption.

The present framework relies on genome SNP distances, and integration of additional genomic features could further enhance discriminatory power. Plasmid content, antimicrobial resistance profiles, and virulence gene distributions may improve epidemiological relationship assessments, particularly for closely related isolates where SNP distance alone provides insufficient resolution. Machine learning approaches incorporating multiple genomic features could enable more nuanced classification than pairwise SNP distance. Notably, pan-genomic analyses of accessory gene content, including genes associated with desiccation tolerance, heavy metal resistance, and Type VI secretion systems, have recently been shown to correlate with *C. sakazakii* isolation source and geographic origin [30], suggesting that accessory genome profiles may complement genome SNP analysis in distinguishing environmental persistence from active transmission and in identifying isolates with elevated pathogenic potential.

Despite these limitations, the framework provides essential infrastructure for enhanced surveillance with immediate public health applications in the United States. The tiered thresholds may support more sensitive identification of putative epidemiological clusters and support broader food safety functions including traceback investigations and root cause analysis. Isolates separated by 100–500 SNPs may nonetheless reveal contamination sources, transmission routes, and environmental persistence patterns that would be invisible to single-threshold approaches, enabling identification of silent contamination events before clinical cases occur.

The documentation of persistent environmental contamination spanning decades, exemplified by the Michigan environmental complex, reveals inadequacies in current decontamination protocols [12,25]. The genetic stability of these populations suggests that established *C. sakazakii* communities are substantially more resilient to standard sanitation measures than previously recognized, necessitating enhanced environmental monitoring and more aggressive intervention strategies [27,57,82,85]. Traditional assumptions of effective decontamination following detection may be insufficient given the pathogen’s demonstrated capacity for long-term facility persistence and biofilm-mediated survival [12,85,94].

## 5. Conclusions

This study presents the largest population genomics analysis of *C. sakazakii* conducted to date, encompassing 1870 genomes and establishing a statistically grounded framework for genomic surveillance of this pathogen. Comprehensive characterization of species-level diversity revealed extensive accessory genome plasticity consistent with *C. sakazakii*’s broad ecological range across food production environments, processing facility surfaces, and clinical infections.

The identification of persistent environmental contamination spanning 24 years, exemplified by the Michigan environmental complex, demonstrates that established *C. sakazakii* populations may be substantially more resilient to standard decontamination protocols than current regulatory frameworks assume.

While not intended as a standalone outbreak-confirmation tool, the tiered framework may support the full spectrum of food safety surveillance applications: traceback investigations, root cause analysis, and detection of silent contamination events prior to clinical case recognition. By formally accommodating the reality that epidemiologically related isolates may be separated by 100–500 SNPs as a consequence of environmental persistence and ongoing microevolution, the framework enables a shift from reactive outbreak response toward proactive risk identification, a shift of particular importance given the severity of *C. sakazakii* infections in premature neonates and other vulnerable populations.

Implementation of this population genomic infrastructure provides an actionable foundation for improving contamination source attribution, strengthening evidence-based regulatory decision-making, and ultimately enhancing protection of vulnerable infant populations through earlier detection and more comprehensive genomic risk assessment. However, as Michigan isolates comprised 39.3% of the dataset, prospective validation across geographically diverse US collections is recommended to confirm the generalizability of the proposed SNP thresholds before broader regulatory adoption.

## Figures and Tables

**Table 1:** Pan-genome partition statistics and gene family distribution.

Partition	Gene Families	Percentage	Description
Total pan-genome	24,035	100.0%	All gene families
Exact core	1033	4.3%	Present in 100% of genomes
Soft core	3437	14.3%	Present in ≥95% of genomes
Shell	3351	13.9%	Accessory genes (3–95%)
Cloud	17,279	71.9%	Rare genes (<3%)

**Table 2:** Comparative pan-genome architecture of select *Enterobacteriaceae* species.

Species	N Genomes	Pan-Genome	Soft Core	Core %	Reference
*K. pneumoniae*	328	29,000	1743	6.0%	[72]
*C. sakazakii*	237	17,158	3346	19.5%	[29]
*C. sakazakii*	748	13,763	3475	25.2%	[30]
*C. sakazakii*	1870	24,035	3437	14.3%	This study
*E. coli*	1324	25,000	3000	12%	[73]
*E. coli*	2377	7580	2398	31.6%	[74]
*S. enterica*	339	15,096	3368	22.3%	[75]
*S. enterica*	4839	25,300	3200	12.6%	[76]

**Table 3:** SNP distance distribution categories and population structure statistics.

Category	SNP Range	Pairs	Percentage	Interpretation
Identical	0	927	0.1%	Duplicates or same strains
Very Close	1–100	24,084	1.4%	Potentially recent transmission/contact
Close	101–500	48,910	2.8%	Potentially epidemiologically related—requires context
Moderate	501–1000	134,366	7.7%	Distant relationships
Distant	1001–5000	92,586	5.3%	Distinct lineages
Very Distant	>5000	1,447,569	82.8%	Highly divergent lineages

**Table 4:** Major phylogenetic clusters.

Cluster ID	No of Genomes	Max SNP Diameter	Bootstrap Support	Dominant Source (%)	Dominant Location (%)	Date Range
1	150	470,347	1.00	Food/Other (27.3%)	Other USA (72.7%)	1970–N/A
2	136	444,562	1.00	Environment (51.5%)	Other USA (52.9%)	2005–N/A
3	124	510,399	0.56	Environment (51.6%)	Other USA (64.5%)	2009–2025
4	111	38,910	1.00	Environment (88.3%)	Michigan (84.7%)	2002–2025
5	105	58,329	0.86	Environment (68.6%)	Michigan (64.8%)	2001–N/A
6	101	8827	0.98	Environment (98.0%)	Michigan (94.1%)	2022–2025
7	84	53,604	1.00	Environment (58.3%)	Other USA (67.9%)	1973–N/A
8	77	26,409	1.00	Environment (42.9%)	Other USA (75.3%)	2002–N/A
9	67	458,578	1.00	Environment (47.8%)	Other USA (61.2%)	2002–N/A
10	64	35,985	1.00	Environment (76.6%)	Other USA (60.9%)	1971–N/A
11	62	40,122	0.96	Inf. Formula (66.1%)	Other USA (75.8%)	2008–2025
12	59	71,151	1.00	Environment (47.5%)	Other USA (62.7%)	2013–2025
13	57	48,771	0.90	Food/Other (29.8%)	Other USA (66.7%)	2004–N/A
14	56	22,522	1.00	Food/Other (37.5%)	Other USA (92.9%)	1970–N/A
15	55	9362	0.89	Environment (100%)	Michigan (100%)	2022–2025
16	53	117,212	1.00	Environment (56.6%)	Other USA (47.2%)	2004–N/A
17	53	16,387	1.00	Environment (100%)	Michigan (100%)	2022–2025
18	52	39,391	1.00	Environment (48.1%)	Michigan (61.5%)	2001–N/A
19	51	81,529	1.00	Inf. Formula (41.2%)	Other USA (86.3%)	2008–2025
20	51	35,326	1.00	Environment (52.9%)	Other USA (54.9%)	2022–N/A
21	51	37,055	1.00	Environment (66.7%)	Michigan (66.7%)	2004–N/A
22	50	5242	0.94	Environment (100%)	Michigan (100%)	2022–2025

Notes: Michigan Environmental Complex clusters (4, 5, 6, 15, 17, 18, 21, 22) comprising 475 genomes (25.4% of dataset) with 24-year temporal persistence (2001–2025). Infant formula-associated clusters showing distinct epidemiological patterns with broad geographic distribution. N/A indicates missing collection year information on some genomes in the cluster.

**Table 5:** Tiered SNP threshold framework and performance validation for *Cronobacter sakazakii*.

Tier	Metadata Criteria Match	Pairs (n)	Median SNPs	95th Percentile Threshold	Sensitivity (%)	FDA Sensitivity	Additional Pairs
1	All 3	110	3	≤234	95	76.4%	20 pairs
2	≥2	169	7	≤506	95	66.3%	48 pairs
3	≥1	209	10	≤498	95	58.9%	76 pairs

Notes: Metadata criteria based on concordance of isolation source, geographic location, and collection year. All three tiers share the same upper range maximum of 1128 SNPs. FDA comparison uses <21 SNP threshold. Additional pairs represent isolates correctly identified by tiered framework, but missed by FDA threshold.

## Data Availability

The original contributions presented in the study are included in the article, further inquiries can be directed to the corresponding author.
